# A Defense of English Dentistry

**Published:** 1886-05

**Authors:** 


### Editorial, Etc.

A DEFENSE OF ENGLISH DENTISTRY.—An editorial in the London *Dental Record* under the title of "An Insult to British Dentistry," exonerates Americans from being the instigators of the following article which appeared in a pamphlet under the name of "A Few Remarks on American Dentistry in England."

"The ordinary English dentists are men who have had no hospital education, in fact no opportunity of seeing thoroughly good operative or artistic dentistry, whose time has been spent in making artificial teeth in perfect rows in the laboratory or work-room of some other dentist, and having saved a little money they start in practice themselves.

"In the two Dental Hospitals in London, which are the best schools for English dentists, thousands of teeth each year are extracted that should and can be saved, which is not only a disgrace to the dentistry of England, but it teaches the young dentist to put no value on their patients' teeth.

"Those who do value their teeth and wish to save them are cautioned to avoid the ordinary English dentists, and to assist in protesting against either having teeth extracted themselves or permitting others to have them extracted."

Commenting upon the above, the *Dental Record* says :

The foregoing paragraphs are downright itsults to English dentists, to teaches of dentistry in England, and to the intelligence of the English people. There is reason to believe that they who are responsible for these gross libels are not Americans. Certainly no one with the least regard for a possible reputation, or for other than purely personal motives, would utter such statements. The article of the *Medical Press* will receive the general approval of the dental profession; but, alas! the unwary public will require many such remedial measures ere it will sing, "Quacks shall no more have dominion over us, but true Heroes and Healers!" The names of the authors of this advertising medium are not to be found in the Dentists' Register.

